# PI3K p110α Isoform-Dependent Rho GTPase Rac1 Activation Mediates H_2_S-Promoted Endothelial Cell Migration via Actin Cytoskeleton Reorganization

**DOI:** 10.1371/journal.pone.0044590

**Published:** 2012-09-07

**Authors:** Li-Jia Zhang, Bei-Bei Tao, Ming-Jie Wang, Hui-Ming Jin, Yi-Chun Zhu

**Affiliations:** Department of Physiology and Pathophysiology, Fudan University Shanghai Medical College, Shanghai, China; Chang Gung University, Taiwan

## Abstract

Hydrogen sulfide (H_2_S) is now considered as the third gaseotransmitter, however, the signaling pathways that modulate the biomedical effect of H_2_S on endothelial cells are poorly defined. In the present study, we found in human endothelial cells that H_2_S increased cell migration rates and induced a marked reorganization of the actin cytoskeleton, which was prevented by depletion of Rac1. Pharmacologic inhibiting vascular endothelial growth factor receptor (VEGFR) and phosphoinositide 3-kinase (PI3K) both blunted the activation of Rac1 and the promotion of cell migration induced by H_2_S. Moreover, H_2_S-induced Rac1 activation was selectively dependent on the presence of the PI3K p110α isoform. Activated Rac1 by H_2_S thus in turn resulted in the phosphorylation of the F-actin polymerization modulator, cofilin. Additionally, inhibiting of extracellular signal-regulated kinase (ERK) decreased the augmented cell migration rate by H_2_S, but had no effect on Rac1 activation. These results indicate that Rac1 conveys the H_2_S signal to microfilaments inducing rearrangements of actin cytoskeleton that regulates cell migration. VEGFR-PI3K was found to be upstream pathway of Rac1, while cofilin acted as a downstream effector of Rac1. ERK was also shown to be involved in the action of H_2_S on endothelial cell migration, but independently of Rac1.

## Introduction

Hydrogen sulfide (H_2_S), along with nitric oxide (NO) and carbon monoxide (CO), is now considered to be the third member of the gaseotransmitter family [1], [2], [3]. H_2_S is endogenously generated from L-cysteine in reactions that are catalyzed by cystathionine-β-synthase (CBS) [4], cystathionine-γ-lyase (CSE) [4], and 3-mercaptopyruvate sulfurtransferase (3-MST) [5], [6]. Recently, H_2_S has been recognized as an important signaling molecule for the nervous system, the cardiovascular system, and the inflammatory system [1], [2], [7]. As a signaling molecule, H_2_S modulates neuronal transmission by facilitating the induction of hippocampal long term potential (LTP) [8]. In the cardiovascular system, H_2_S has been shown to relax smooth muscles and to regulate blood pressure [9], [10], [11]. H_2_S can act as a cytoprotectant to protect cells from oxidative stress and as an anti-apoptosis agent by preserving mitochondrial function during ischemia-reperfusion [12], [13]. H_2_S has also been found to regulate the release of insulin [14], [15]. Both pro-inflammatory and anti-inflammatory effects of H_2_S have recently been discovered also [16], [17]. While in our previous study, we have provided the first piece of evidence regarding the proangiogenic effect of exogenous H_2_S in a matrigel plug model [18]. And our subsequent study has also verified the proangiogenic effect of H_2_S in a rat model of hind limb ischemia [19]. Researches by Papapetropoulos et al also indicate that H_2_S is an endogenous stimulator of angiogenesis [20]. However, the concrete underlying molecular mechanisms of H_2_S on angiogenesis are still poorly understood.

Vascular endothelial cell migration has a pivotal role in angiogenesis. A series of cellular events including changes in the actin cytoskeleton, in cell-matrix adhesions, cell-cell interaction, and in the biosynthesis and degradation of extracellular matrix are involved in cell migration [21]. Cell migration is characterized with actin polymerization in the protruding front edges of the cells and retraction of the cell tail ends [22]. The migrating cells are driven with the mechanical force provided by dynamic remodeling of the actin cytoskeleton which is downstream of the intracellular signaling pathways involving the small GTPases of the Rho family, in particular Rac1, RhoA and Cdc42 [23]. However, it is not known if small Rho GTPases play a role in mediating the action of H_2_S in angiogenesis and migration of vascular endothelial cells.

The aim of this study was to test the hypothesis that small Rho GTPases mediate the action of H_2_S on endothelial cells. Using multifaceted approaches, we provide the first evidence that H_2_S promotes the migration of human endothelial cells through Rho GTPase Rac1-mediated actin cytoskeleton reorganization. In addition, the upstream regulators and downstream effectors of Rac1 were also particularly monitored. An important physiological role of H_2_S as an endothelial cell migration promoting factor and the detailed signaling transduction pathway of H_2_S are thus elucidated.

## Experimental Procedures

Materials-Fetal bovine serum (FBS) and trypsin were obtained from Gibco (Carlsbad, CA, USA). Antibodies against paxillin, ERK, p38, JNK, Akt, GAPDH, cofilin, GFP, p110α, p110β and p110γ were purchased from Cell Signaling Technology (Beverly, MA, USA). Antibodies against actin, p110δ and HA were purchased from Santa Cruz Biotechnology (CA, USA). The antibody against paxillin that was used for immunofluorescence was purchased from Abcam (Cambridge, UK). Rho GTPases pull down activation assay kits and antibodies against Rac1, Cdc42, and RhoA were from Millipore (Temecula, CA, USA). All of the pharmacologic inhibitors such as LY 294002, U0126, NSC23766, and Su5416 were obtained from Tocrics (Bristol, UK). Growth factor reduced matrigel and human recombinant VEGF were from BD Biosciences (Bedford, MA, USA). Gelatin and NaHS were from Sigma (St Louis, MO, USA). Human recombinant Rac1 and G-LISA Rac1 activation assay kits were obtained from Cytoskeleton (Denver, CO, USA).

Cell culture-Primary human umbilical vein endothelial cells (HUVECs) were obtained from AllCells (Emeryville, CA, USA). Each batch of HUVECs was examined using flow cytometry and was shown to be 99% CD31 positive, with a viability of 95%. Cells were cultured in complete HUVECs medium on plates coated with 0.5% gelatin. HUVECs (passage 3 or 4) that were approximately 70% confluent were used for most experiments. Cells were serum starved in 0.1% fetal bovine serum in endothelial cell basal medium for 24 h prior to the various treatments. Cells were passaged 1 day prior to electroporation to ensure that they were actively growing on the day of electroporation.

Monolayer wound healing assay and transwell boyden chamber assay-For the monolayer wound healing assay, confluent HUVECs were starved for 24 h before starting the experiments. The cell monolayer was wounded by scratching with a plastic pipette and was washed three times with PBS. Images were taken at 0 h or 6 h after wounding, and the wound closure was calculated 6 h later.

The transwell migration assays were performed as previously described with little modification [18]. Briefly, cells (5×10^4^/well) were plated on 8 mm pore transwell filters (Corning, Lowell, MA, USA) that were coated with 0.5% gelatin. After 16 hours, the filters were fixed with 4% formaldehyde and stained with 0.4% crystal violet in 10% ethanol. Non-migrated cells on the upper side of the filters were gently removed using a cotton swab, and cells on the underside of the filters were photographed. To quantify cell motility, cells that stained positively with crystal violet in nine random fields of each filter were counted, and three independent filters were analyzed.

In vitro endothelial tube formation assay-Growth factor-reduced matrigel matrix was plated evenly in a 24-well plate (200 µl/well) and incubated at 37°C for 30 min before adding HUVECs (75,000 cells/well). Then, 16 h after seeding, photographs of representative 10× fields were taken (*n*≥5 per condition and genotype), and endothelial tubes were quantified by counting branches and tube length.

Plasmids-pcDNA3-EGFP-Rac1-T17N (Addgene plasmid 12982) as the dominant negative plasmid of human Rac1 gene and pcDNA3-EGFP (Addgene plasmid 13031) as the empty control vector were both obtained from the plasmid repository at Addgene (Cambridge, MA, USA). Hemagglutinin (HA)-tagged dominant-negative (DN) Akt (kinase-inactive mutant Myr-Akt-K179M) [24] was a kind gift from Dr. Jin Q. Cheng (Department of Pathology and Interdisciplinary Oncology, University of South Florida College of Medicine, H. Lee Moffitt Cancer Center, Tampa, Florida). All the plasmids were confirmed by sequencing before amplification. Since HUVECs are hard to transfect, electroporation as described below was used to transfect the cells.

RNA interference-All small interfering RNAs (siRNAs) were synthesized by Ambion (Austin, TX, USA). Oligonucleotide sequences were as follows: Rac1 siRNA (sense: 5′-GGAACUAAACUUGAUCUUAtt-3′, antisense: 5′-UAAGAUCAAGUUUAGUUCCca-3′), p110α siRNA (sense: 5′-GUAAUUACCCAGAUCCUAUtt-3′, antisense: 5′-AUAGGAUCUGGGUAAUUACag-3′), p110β siRNA (sense: 5′-GGGAAAGCUGGACUACUAAtt-3′, antisense: 5′-UUAGUAGUCCAGCUUUCCCtg-3′), p110γ siRNA (sense: 5′-GCUUUAGAGUUCCAUAUGAtt-3′, antisense: 5′-UCAUAUGGAACUCUAAAGCtt-3′) and p110δ siRNA (sense: 5′-GACUAAUAAUAGUGAGAAAtt-3′, antisense: 5′-UUUCUCACUAUUAUUAGUUCtt-3′). For RNA interference experiments, HUVECs were electroporated with scrambled siRNA as a negative control (Ambion), GAPDH siRNA (Ambion) as a positive control and each of the targeting silencer oligonucleotides.

Electroporation-Prior to electroporation, the cells were washed with PBS, counted, and resuspended in Gene Pulser electroporation buffer (Bio-Rad, Hercules, CA, USA) to a density of 1×10^6^ cells/ml, and mixed with nucleic acid. A fluorescently labeled transfection control siRNA (Ambion) was used to assess the electroporation efficiency and to screen for electroporation parameters. The plasmids were used at a concentration of 20 µg/ml, and silencer siRNAs were used at 100 nM. The cells and nucleic acid were gently mixed in Gene Pulser electroporation buffer, and 600 µl of the mix was transferred into 4 mm electroporation cuvette (Bio-Rad). Electroporation was carried out using a manually entered protocol (250 V, 10 ms) on the Gene Pulser Xcell electroporation system (Bio-Rad). The electroporated cells (200 µl) were transferred into a 6-well tissue culture plate containing 2 ml of growth media/well and were incubated at 37°C for 24 h, unless otherwise indicated.

Analysis of transfection-Cells that were electroporated with EGFP-containing plasmids or fluorescent control siRNA were observed and photographed under a fluorescence microscope 48 h after electroporation. Delivery of silencer siRNA was also assessed by reverse transcription-quantitative polymerase chain reaction (RT-qPCR). Total RNA was extracted from cells using an RNArose reagent, and cDNA was synthesized using the ReverTra Ace qPCR RT Kit (Toyobo, Osaka, Japan). Gene-specific primers were used to amplify relevant messages using SYBR Green Master Mix (Toyobo) on an iCycler iQ™ real-time PCR detection system (Bio-Rad). All RT-qPCR reactions were carried out in triplicate.

For the Rac1 gene, the forward primer was 5′-GCCAATGTTATGGTAGAT-3′ and the reverse primer was 5′-GACTCACAAGGGAAAAGC-3′. For the GAPDH gene, the forward primer was 5′-AACGGATTTGGTCGTATTG-3′ and the reverse primer was 5′-GCTCCTGGAAGATGGTGAT-3′. For the actin gene, the forward primer was 5′-CACCAACTGGGACGACAT-3′ and the reverse primer was 5′-ACAGCCTGGATAGCAACG-3′.

Immunofluorescence and confocal microscopy-HUVECs grown on coverslips were fixed by 4% paraformaldehyde in PBS for 30 min, permeabilized in 0.1% Triton X-100 and blocked in PBS containing 2% BSA for 1 h at room temperature. The samples were incubated with primary antibodies overnight at 4°C, followed by incubation with Alexa Fluor-labeled secondary antibody (Invitrogen, Carlsbad, CA, USA) for 1 h at room temperature. The cells were washed 3 times with PBS and stained for F-actin and DNA with Rhodamine-phalloidin (Invitrogen) and DAPI solution, respectively, in dark chamber. After washing with 0.05% Tween 20-PBS, the specimens were mounted in Fluoromount Aqueous mounting medium (Sigma). Confocal laser scanning microscopy was carried out with a Leica TCS SP5 confocal microscope (Wetzlar, Germany) using an oil immersion objective.

Pull-down assay: measurement of Rho GTPases activity-The ability of Rac1-GTP and Cdc42-GTP to bind GST-PAK1-PBD (p21-activated kinase-binding domain) and the ability of RhoA-GTP to bind to GST-Rhotekin-RBD (Rho binding domain) were used to determine the cellular levels of active GTPases. Small Rho GTPases GTP pull-down assays were performed by incubating the cleared lysates with glutathione agarose beads that were coupled to GST-PAK1-PBD or GST-Rhotekin-RBD (Millipore) for 1 h at 4°C. The beads were then followed by four washes in washing buffer. Bound proteins were solubilized by the addition of 25 µl of SDS-PAGE Laemmli loading buffer and separated by 12% SDS-PAGE, followed by western blotting for Rac1, Cdc42 or RhoA proteins.

G-LISA (ELISA-based GTPase activation assays): measurement of the GTP-bound form of Rac1-After protein lysates being collected, the activated GTP-bound Rac1 was analyzed with a G-LISA activation assay biochemistry kit (Cytoskeleton, Denver, CO, USA) according to the manufacturer’s instructions.

In vitro reaction assay between Rac1 and H_2_S-To detect the direct action of H_2_S on Rac1, we used an in vitro reaction system. GTP (200 µM) and different concentrations of NaHS were respectively added into 50 µl reaction buffer (25 mM HEPES, pH 7.5, 150 mM NaCl, 1% lgepal CA-630, 10 mM MgCl_2_, 10 mM EDTA and 2% glycerol) that contained 20 ng human recombinant Rac1 (Cytoskeleton). We used GDP (200 µM) instead of GTP as a negative control and GTPγS (200 µM) instead of GTP as a positive control separately in the reaction system. After incubation at 30°C for 30 min, the reaction was stopped, and the activated form of Rac1 was assayed using a G-LISA assay kit (Cytoskeleton) in accordance with the manufacturer’s instructions.

Statistical analysis-Quantitative data are presented as the means ± SE. Differences between groups were analyzed using one-way ANOVA followed by post-hoc Tukey’s test, where applicable. In all cases, a *P* value of <0.05 was taken to indicate statistical significance.

## Results

**Figure 1:**
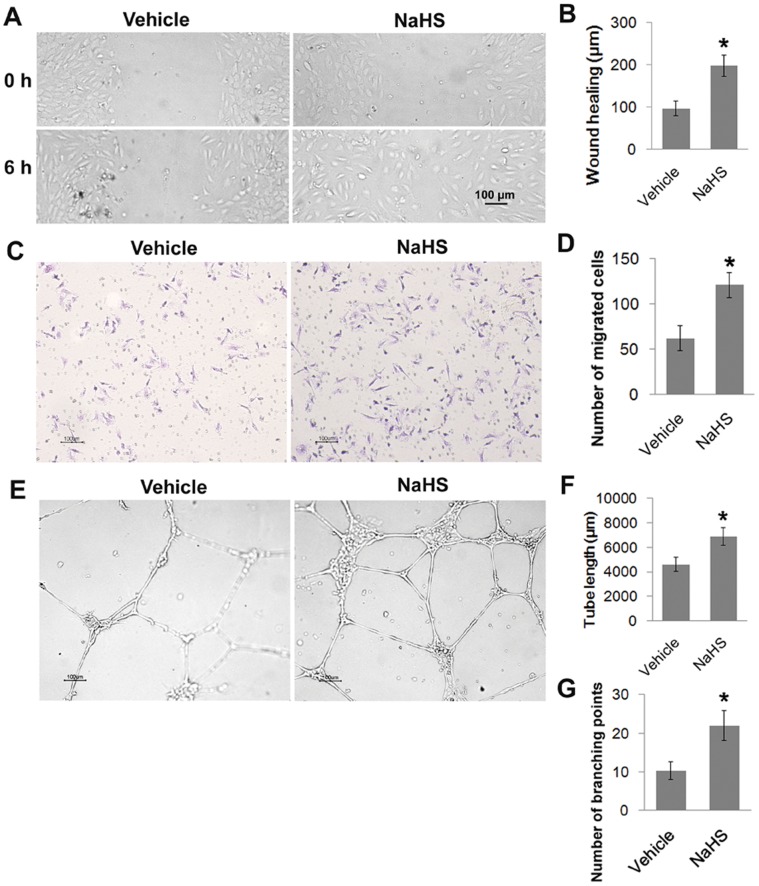
H_2_S promotes endothelial cell migration and microvessel tube formation. (A) Representative micrographs of monolayer wounding assay in HUVECs treated with or without NaHS (50 µM) at 0 h and 6 h after treatment. (B) Statistical analysis of the scratch wounding assay. (C) Cell migration was also assessed by transwell boyden chamber assay. Shown are the representative micrographs and the values (D) of the migrated cells treated with 50 µM NaHS. (E) Representative micrographs of microvessel tube formation in HUVECs treated or not treated with NaHS (50 µM). Statistical analysis of tube length (F) and branching points (G). Data represent the means ± SE of five independent experiments. Each experiment was performed in duplicate. *P<0.05.

H_2_S promotes cell migration and microvessel tube formation in human endothelial cells-To test the action of H_2_S on HUVECs, we used sodium hydrogen sulfide (NaHS) as a precursor for H_2_S. Exposure of HUVECs to 50 µM NaHS promoted an increase in the cell migration rate with an approximate 2-fold increase in both the scratch wounding assay (NaHS vs. Vehicle: 197.8±24.4 µm vs. 96.6±17.5 µm; *P*<0.05; Fig. 1A, B) and the transwell boyden chamber assay (NaHS vs. Vehicle: 121±14 vs. 62±14; *P*<0.05; Fig. 1C, D). Furthermore, 50 µM NaHS significantly enhanced capillary-like structure formation of HUVECs cultured on reduced-growth factor matrigel, which was reflected by both of tube length (NaHS vs. Vehicle: 6902.6±717.2 µm vs. 4592.7±567.2 µm; *P*<0.05; Fig. 1E, F) and number of tube branches (NaHS vs. Vehicle: 21.9±3.9 vs. 10.3±2.3; *P*<0.05; Fig. 1E, G).

**Figure 2:**
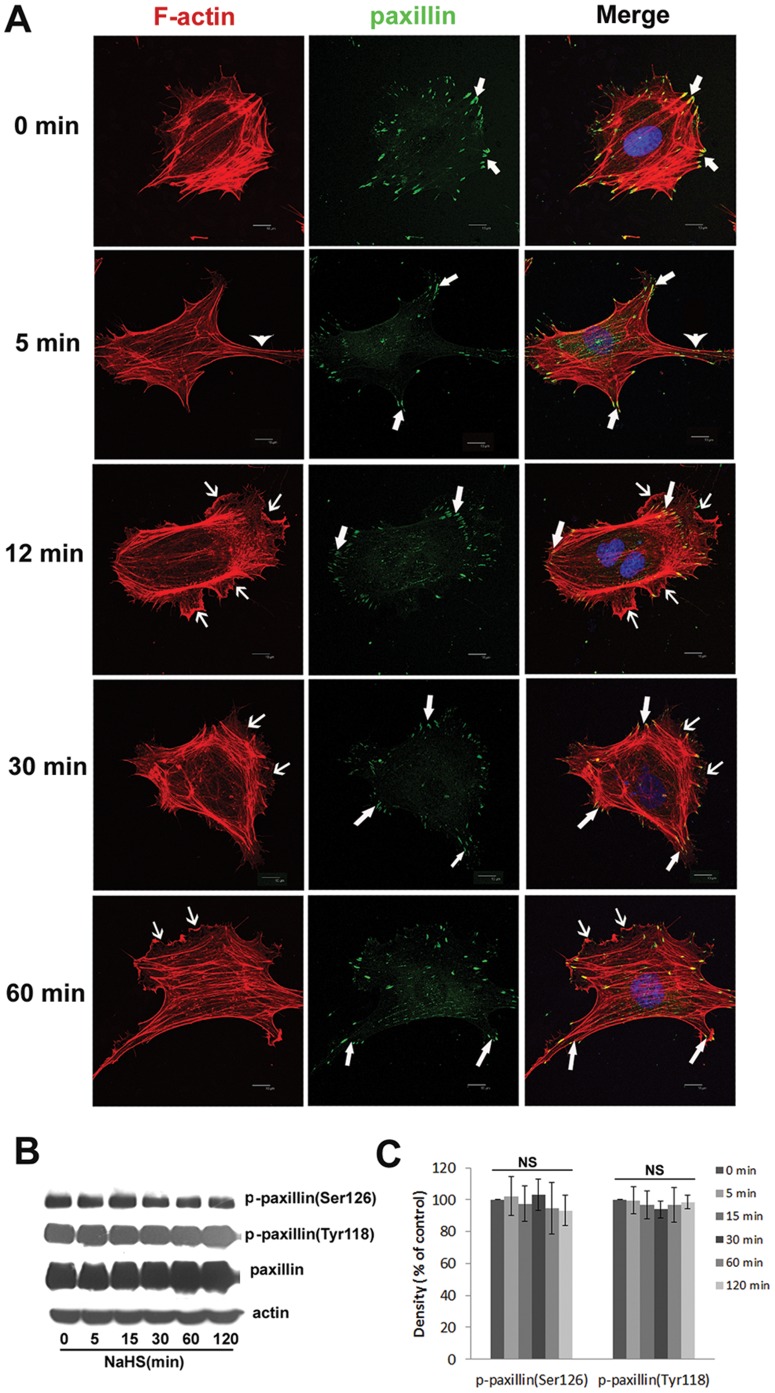
H_2_S induces reorganization of the actin cytoskeleton in HUVECs. (A) The effects of H_2_S on the actin cytoskeleton and on paxillin-containing focal adhesions. Serum-starved HUVECs were stimulated or not by 50 µM NaHS for indicated times. Thereafter, immunofluorescence analysis was performed using DAPI (blue) to stain nucleus, rhodamine-conjugated phalloidin (red) to stain F-actin and a mouse anti-paxillin monoclonal antibody followed by Alexa Fluor-labeled secondary antibody (green) to stain paxillin-contained focal adhesions. An overlay of fluorescent signals, generating yellow color in areas of colocalization, is shown on the right column. The thin arrows indicate lamellipodia, arrowheads indicate cell protrusions and thick arrows show paxillin-containing focal adhesions. Scale bars, 10 µm. Representative blots (B) and statistical values (C) showing that NaHS (50 µM) treatment has no effect on the phosphorylation of paxillin. The results are representative of three independent experiments. Values are means ± SE. NS, not significant.

H_2_S induces actin cytoskeleton reorganization in endothelial cells-As shown with immunostaining of F-actin (Fig. 2A), an endothelial cell without NaHS treatment appeared spindle-shaped with regular paralleled actin filaments across the cell body. The stress fibers were distributed evenly in the cortical region of the cell. In contrast, in cells treated with NaHS, there were significant changes regarding the cellular actin cytoskeleton. We observed that after 5 min exposure to 50 µM NaHS, the endothelial cell formed protrusions in the lateral body of the cell (depicted by an arrowhead in Fig. 2A). Following prolonged NaHS treatment, the typical lamellipodia were generated most prominently in the leading cell edge (depicted by a thin arrow in Fig. 2A) and the characteristically shaped membrane ruffles were apparent. As shown in Fig. 2A, the NaHS-induced augmentation of typical lamellipodia in the leading cell edge indicates the reorganization of the actin cytoskeleton.

As depicted in Fig. 2A, paxillin is specifically localized to focal adhesions, which are used to anchor stress fibers (depicted by the thick arrow in Fig. 2A). Compared with control cells, treatment with NaHS did not change the localization of paxillin. The paxillin-containing focal adhesion had no evident turnover transmission or redistribution after NaHS treatment. In addition, the phosphorylation of paxillin on Ser126 and Tyr118 did not change (Fig. 2B).

**Figure 3:**
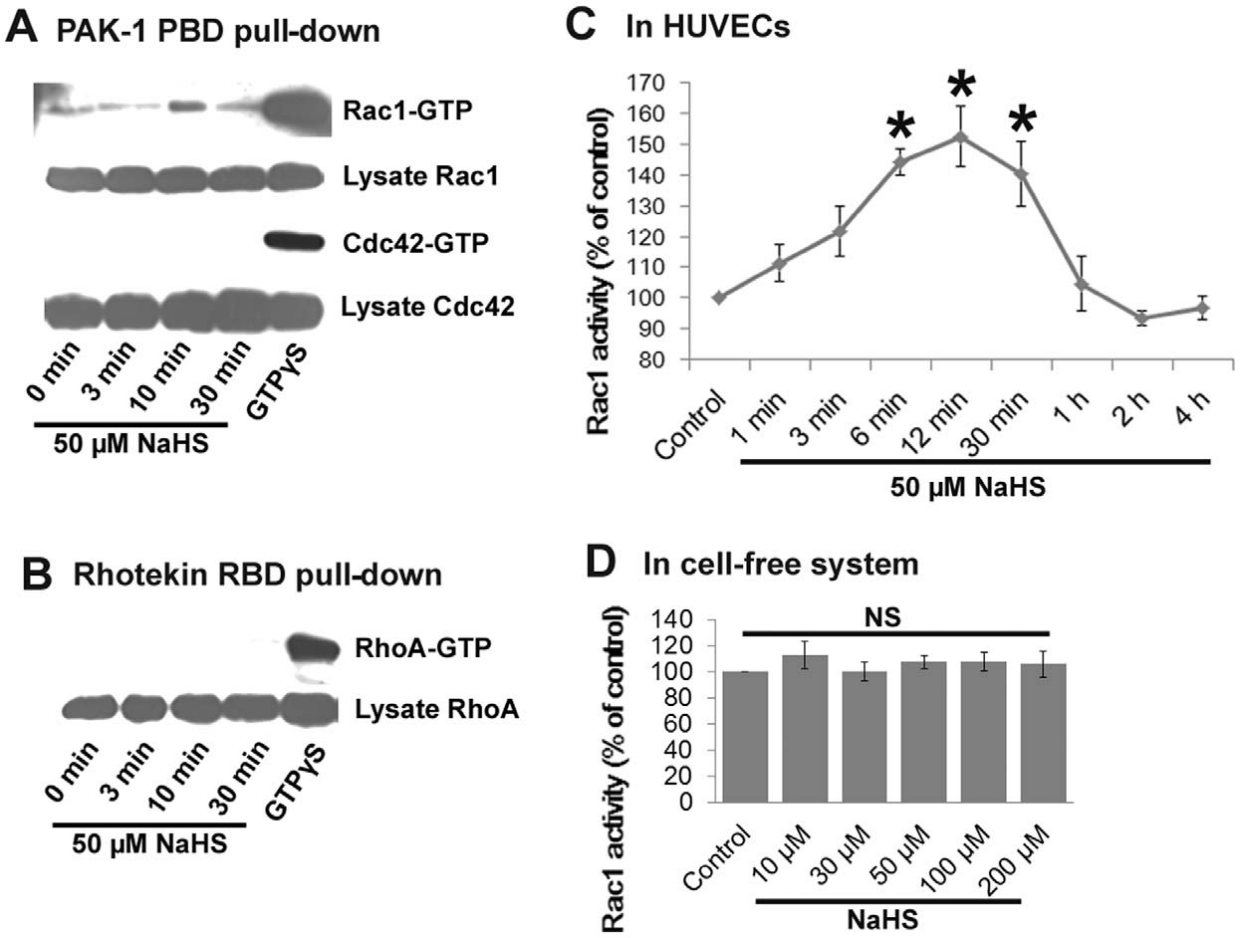
H_2_S selectively activates the Rho GTPase Rac1 in HUVECs but does not directly activate Rac1 in vitro. (A, B) Pull down assays were conducted to detect the activity of Rho GTPases. Serum-starved HUVECs were treated with 50 µM NaHS for indicated times. Cells were then lysed and incubated with GST-PAK1-PBD-bound or GST-Rhotekin-RBD-bound glutathione beads. GTP-loaded Rac1 and Cdc42 bound to GST-PAK1-PBD (A) and GTP-loaded RhoA bound to GST-Rhotekin RBD (B) were detected by western blotting with antibodies against Rac1, Cdc42 or RhoA (upper panel). GTPγS loaded as positive control. Total cell lysates were also probed with the same antibodies to demonstrate that equal amounts of total protein were used in individual assays (lower panel). The results are representative of three independent experiments. (C) The time course for Rac1 activation by H_2_S. ELISA-based GTPase activation assays (G-LISA) of Rac1 were performed at various times after treatment with 50 µM NaHS. (D) In vitro interaction of H_2_S with Rac1. Human recombinant Rac1 was allowed to react with different concentrations of NaHS, and G-LISA assays were performed to detect the level of GTP-bound Rac1. The data are presented as means ± SE of three independent experiments each performed in triplicate. *P<0.05 vs control. NS, not significant.

H_2_S selectively activates the Rho GTPase Rac1 in HUVECs-The results from pull-down assays demonstrated that Rac1 was quickly activated after treatment with NaHS for 10 min (Fig. 3A), whereas RhoA and Cdc42 were not activated by NaHS (Fig. 3A, B). To verify that Rac1 was activated by NaHS, we further used a G-LISA assay. The data indicate that Rac1 was activated by 50 µM NaHS in a time-dependent manner and reached a peak at 12 min (Fig. 3C). It should be noted that the activation of Rac1 was prompt and temporary; a three- min treatment with NaHS induced activation of Rac1, which declined rapidly to normal after 30 min of treatment.

H_2_S does not directly activate Rac1 in vitro-The purified recombinant Rac1 was allowed to react with different concentrations of NaHS varying from 0–200 µM. G-LISA assay was conducted to detect the activity of Rac1 after each reaction. The results showed no significant difference between groups (Fig. 3D), revealing that H_2_S did not interact with Rac1 in the in vitro cell-free system. Together, these results suggest that Rac1 may be activated by an upstream molecule that is directly activated by H_2_S, which in turn stimulates Rac1.

**Figure 4:**
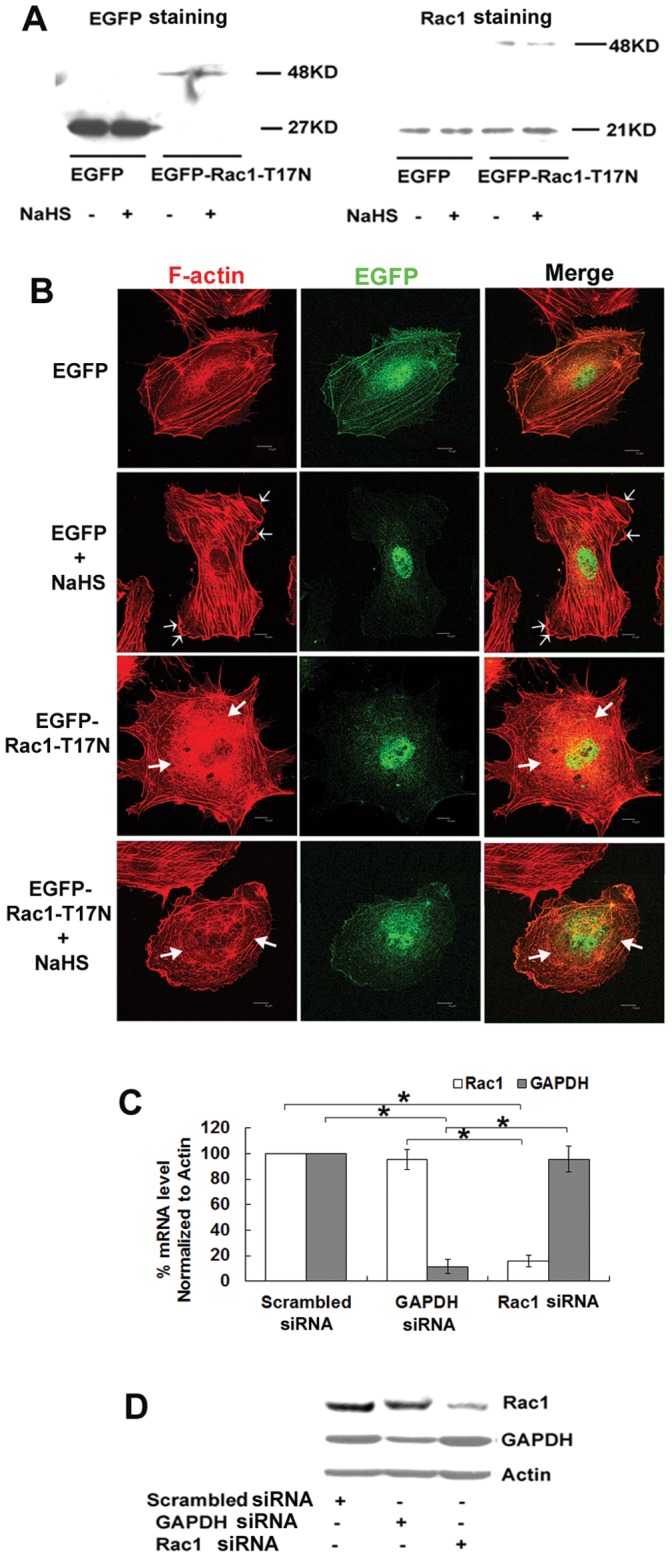
Dominant negative Rac1 inhibits the H_2_S-induced reorganization of the actin cytoskeleton in HUVECs. (A) HUVECs were transfected with plasmids encoding EGFP only (EGFP) or dominant-negative Rac1 (EGFP-Rac1-T17N). Western blot analysis showed manifest levels of the EGFP-Rac1 fusion protein in HUVECs transfected with EGFP-Rac1-T17N vector but not in the cells transfected with EGFP control vector. (B) Transfected cells were starved and then treated with or without 50 µM NaHS for 12 min. Cells were then fixed and stained with rhodamine-labeled phalloidin for F-actin and anti-EGFP antibody for EGFP-tagged Rac1. Representative images are shown. Thin arrows point to the formed lamellipodia. Thick arrows indicate the fragments of disrupted F-actin around the nucleus. Scale bars, 10 µm. (C, D) Rac1 siRNA decreased the expression of Rac1 both on the mRNA levels (C) and protein levels (D). (C) Electroporation was used to transfect HUVECs with siRNA. 48 h after transfection, mRNA transcripts of Rac1 and GAPDH as measured by real-time PCR were significantly reduced by siRNA separately. (D) Representative blots were shown on the protein levels after 72 h transfection. Transfection of HUVECs with Rac1 siRNA and GAPDH siRNA specifically knocked down the expression of their respective target genes without affecting each other’s targets. Values represent the means ± SE, *n* = 9. *P<0.05.

Rac1 mediates H_2_S-induced lamellipodia formation-The EGFP-Rac1-fusion protein was expressed in HUVECs 48 hours after transfection with EGFP-Rac1-T17N but was not expressed in cells that were transfected with the EGFP control vector (Fig. 4A). Examination of the actin cytoskeleton indicated that the H_2_S-mediated induction of the typical wavy lamellipodia (indicated by a thin arrow in Fig. 4B) at the cell edge was inhibited by the presence of dominant negative Rac1. Moreover, fragments of disrupted actin filaments (indicated by a thick arrow in Fig. 4B) in the cell body were observed in cells that were transfected with dominant negative Rac1.

**Figure 5:**
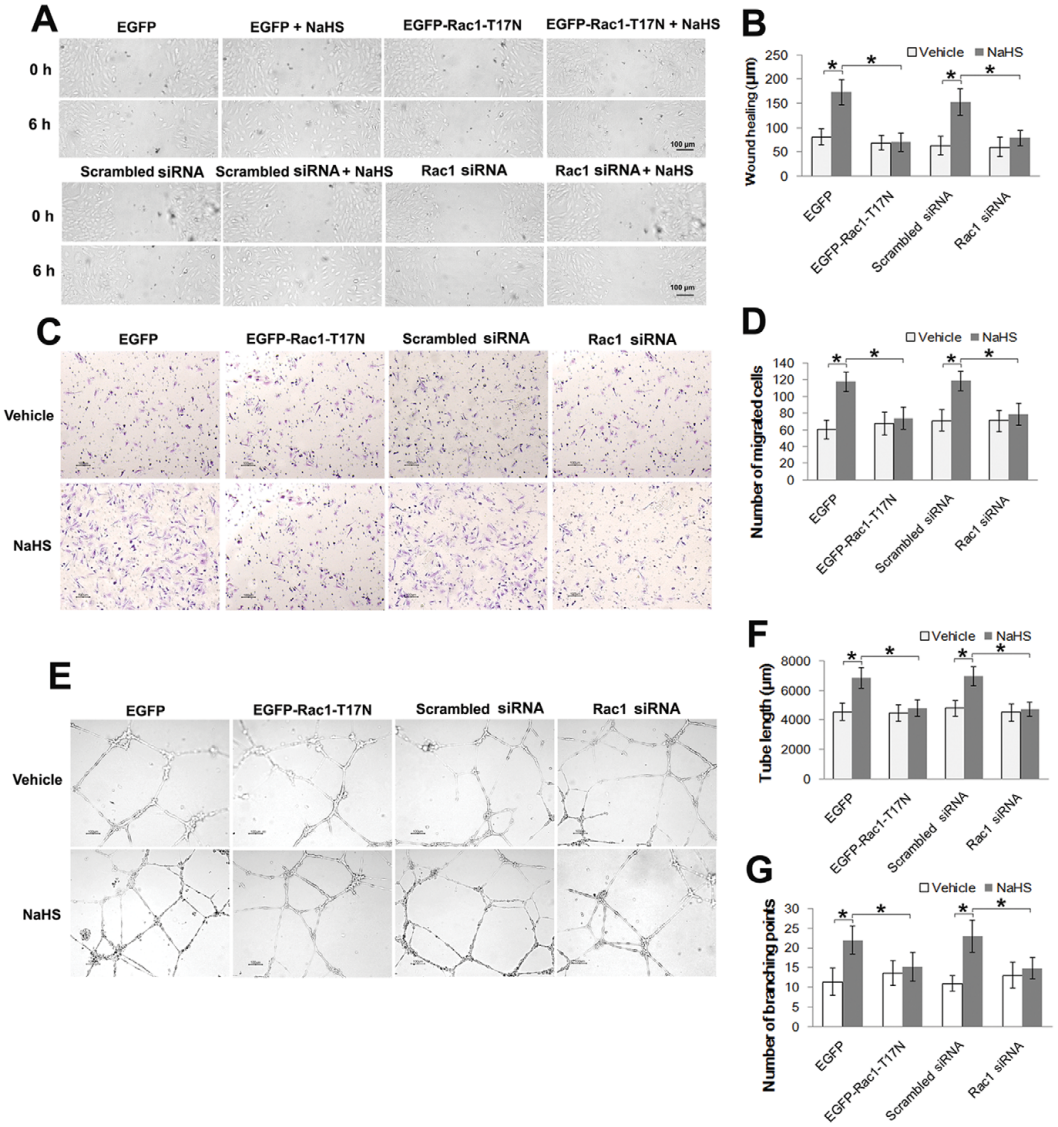
Dominant negative Rac1 and Rac1 siRNA both decrease H_2_S-promoted cell migration and tube formation in HUVECs. Representative micrographs (A) and statistical analysis (B) of scratch wounding assays showing that expression of dominant-negative Rac1 (T17N) and Rac1 siRNA reduced the cell migration rate promoted by 50 µM NaHS. Representative graphs (C) and statistical data (D) of migrated cells in transwell boyden chamber assay indicating that dominant-negative Rac1 (T17N) and Rac1 siRNA affected the cell migration promoted by 50 µM NaHS. Representative pictures (E) and statistical data of tube length (F) and branching points (G) manifesting that dominant-negative Rac1 (T17N) and Rac1 siRNA blunted the microvessel tube formation in three-dimensional culture. Data represent the means ± SE of five independent experiments. Each experiment was performed in duplicate. *P<0.05.

H_2_S-induced endothelial cell migration and microvessel tube formation are both dependent on Rac1-Transfection of Rac1 siRNA and GAPDH siRNA (positive control) specifically knocked down the expression of their respective target genes (Fig. 4C) and proteins (Fig. 4D). As shown in Fig. 5, compared to the EGFP control vector group, dominant negative Rac1 significantly blunted the cell migration effect caused by the 50 µM NaHS treatment both in the scratch wounding assay (Fig. 5A, B) and the transwell boyden chamber assay (Fig. 5C, D). Similarly, Rac1 siRNA induced a marked reduction in the promoted migration rate that was induced by NaHS (Fig. 5A, B, C, D). In the tube formation assay, both the dominant negative Rac1 and Rac1 siRNA significantly blunted vessel-like structure formation after treatment with NaHS (Fig. 5E, F, G).

**Figure 6:**
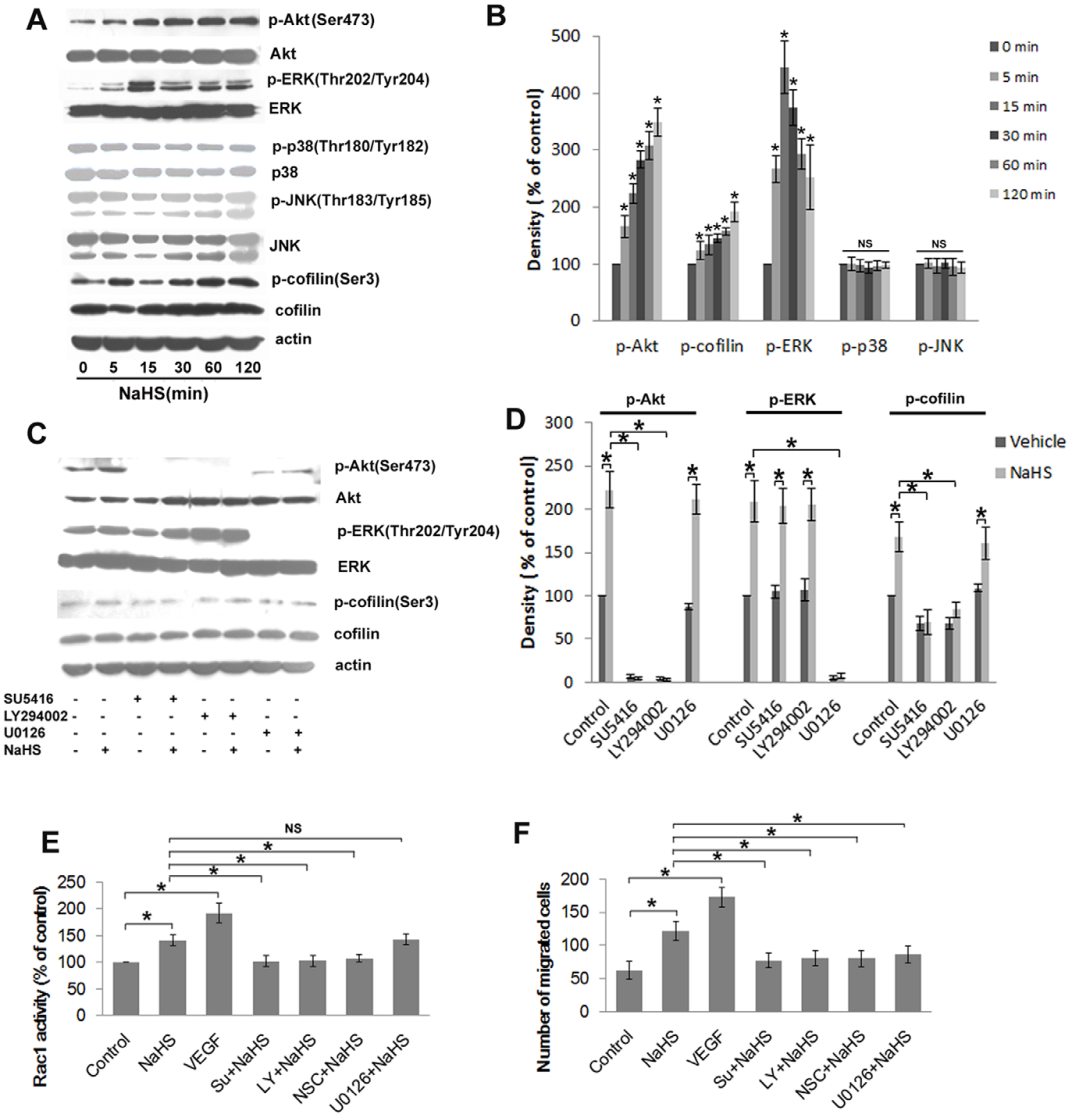
H_2_S signals through VEGFR-PI3K pathway to mediate the activation of Rac1 and to promote cell migration of HUVECs. Representative blots (A) and statistical analysis (B) showing the effects of NaHS (50 µM) treatment on the phosphorylation of Akt, cofilin and MAPKs including ERK, p38 and JNK. (C) The action of pharmacologic inhibiting on the increased phosphorylation of Akt, ERK and cofilin triggered by NaHS and the respective statistical data (D). (E) G-LISA assays measuring the effect of recombinant VEGF (10 ng/ml) and different chemical inhibitors, including SU5416, LY294002, U0126 and NSC23766, on the activation of Rac1. (F) Statistical data of transwell boyden chamber assay showing that the promotion effect of NaHS on endothelial cell migration was inhibited by SU5416, LY294002 and U0126. VEGFR inhibitor-SU5416 (5 µM), PI3K inhibitor- LY294002 (10 µM), MEK inhibitor- U0126 (10 µM), Rac1 inhibitor- NSC23766 (50 µM). Data represent the means ± SE. *P<0.05. NS, not significant.

H_2_S signals through the VEGFR-PI3K pathway to mediate the activation of Rac1 and to promote cell migration in HUVECs-HUVECs that were exposed to 50 µM NaHS exhibited a sustained increase in Akt phosphorylation that was evident as early as 5 min and that lasted up to 2 h (Fig. 6A, B) post treatment. Moreover, treatment with 50 µM NaHS led to ERK (Fig. 6A, B) and cofilin phosphorylation (Fig. 6A, B), albeit with different kinetics, e.g., phosphorylation of ERK was rapid and transient, whereas cofilin phosphorylation showed a delayed but more sustained pattern. With the exception of ERK, there was no obvious change in the phosphorylation levels of two other MAPKs members, p38 and JNK (Fig. 6A, B). Inhibiting VEGFR using SU5416 or PI3K (LY294002) completely abrogated the NaHS-induced phosphorylation of Akt (Fig. 6C, D), which demonstrates that H_2_S signals through the VEGFR-PI3K pathway in HUVECs. The observation that both SU5416 and LY294002 have no effect on the NaHS-induced activation of ERK (Fig. 6C, D) signifies that ERK acts independently of the VEGFR-PI3K pathway. Both SU5416 and LY294002 could abolish the enhanced phosphorylation of cofilin by NaHS (Fig. 6C, D).

Compared to NaHS treatment, VEGF induced a more pronounced augmentation of Rac1 activity (NaHS vs. VEGF: 140.9±10.2% vs. 191.4±18.3% Fig. 6E). Inhibition of Rac1 by NSC23766 significantly reduced NaHS-induced Rac1 activation, and the inhibition of VEGFR with SU5416 significantly reduced H_2_S-induced activation of Rac1 (Fig. 6E). Similar results were obtained using specific inhibitors of PI3K (LY294002) (Fig. 6E), whereas pretreating with the MEK inhibitor U0126 had no effect on Rac1 activation by H_2_S (Fig. 6E). These results suggest that H_2_S-induced Rac1 activation is regulated by the VEGFR-PI3K pathway and that the activation of Rac1 triggered by H_2_S is independent of the MEK-ERK pathway.

Moreover, the cell migration rate promoted by NaHS (50 µM) was also significantly blunted by the VEGFR inhibitor SU5416 and the PI3K inhibitor LY294002 (Fig. 6F). Interestingly, the MEK inhibitor U0126 also has an attenuating effect on cell migration induced by H_2_S (Fig. 6F).

**Figure 7:**
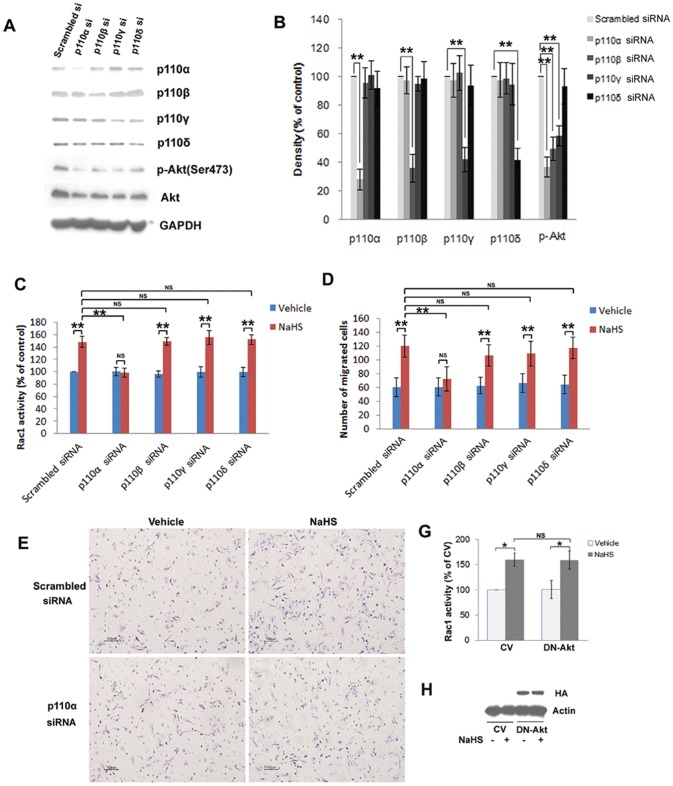
The p110α isoform of PI3K is required for H_2_S-induced Rac1 activation and endothelial cell migration. (A) HUVECs transfected with p110α, p110β, p110γ, p110δ or scrambled siRNAs were lysed and analyzed by immunoblotting for PI3K subunits and for Akt phosphorylation on site of Ser473. SiRNA oligonucleotides specifically knocked down expression of their target genes, and GAPDH was used as a loading control. (B) Densitometric analysis of three independent immunoblots to assess Akt phosphorylation levels and the levels of the p110 isoforms following the knockdown experiments. (C) G-LISA assays for Rac1 activation following the action of different p110 isoforms. 50 µM NaHS-induced Rac1 activation was prevented by p110α siRNA. Statistical data (D) and representative micrographs (E) of transwell boyden chamber assays showing that the promotion effect of NaHS on endothelial cell migration was inhibited by p110α siRNA. (G) G-LISA assays for Rac1 activation were also performed following the expression of dominant negative Akt (DN-Akt). Data showing that NaHS (50 µM) induced Rac1 activation cannot be prevented by DN-Akt. (H) The expression of the HA-tag protein demonstrates that cells were successfully transfected. CV, control vector. Data represent the means ± SE. **P<0.01. *P<0.05. NS, not significant.

The PI3K p110α isoform is required for H_2_S-induced Rac1 activation and endothelial cell migration-H_2_S-induced Rac1 activation is dependent on the VEGFR-PI3K pathway. As the direct downstream effector of PI3K, Akt can be phosphorylated on residue Ser473 by H_2_S treatment (Fig. 6A). As shown in Fig. 7G, the observation that dominant negative Akt has no effect on the H_2_S-induced elevated activity of Rac1 indicates that H_2_S-induced Rac1 activation is independent of Akt. The successful transfection of dominant negative Akt was confirmed by western blot analysis using antibodies to the HA-tag (Fig. 7H).

Knockdown of the individual PI3K isoforms did not affect the expression levels of the other isoforms (Fig. 7A, B). Knockdown of p110α, p110β, and p110γ significantly reduced Akt phosphorylation at Ser473 (Fig. 7A, B). The strongest reduction was observed after p110α depletion, whereas p110δ siRNA had only a small effect on Akt phosphorylation.

As shown in Fig. 7C, depletion of the p110α isoform using p110α siRNA significantly blunted the H_2_S-induced activation of Rac1, whereas p110β siRNA, p110γ siRNA and p110δ siRNA had little effect on the activated Rac1. Moreover, in the transwell boyden chamber assay, only the deletion of p110α markedly prevented H_2_S-induced endothelial cell migration (Fig. 7D, E).

**Figure 8:**
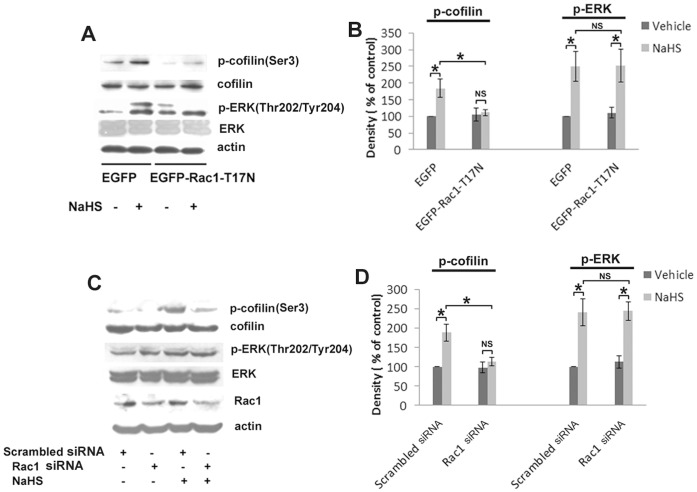
Dominant negative Rac1 and Rac1 siRNA abolish the phosphorylation of cofilin initiated by H_2_S, but have no effect on that of ERK. Representative blots (A) and statistical analysis (B) showing the effects of dominant negative Rac1 on the phosphorylation of cofilin (Ser3) and ERK (Thr202/Tyr204). (C) Representative blots and statistical analysis (D) showing Rac1 siRNA on the phosphorylation of cofilin (Ser3), ERK (Thr202/Tyr204). Values represent means ± SE; *n* = 3 in each group. *P<0.05. NS, not significant.

Cofilin, but not ERK, acts as the downstream effector of Rac1-HUVECs exposed to 50 µM NaHS exhibited a marked increase in cofilin phosphorylation in a time-dependent manner (Fig. 6A). Dominant negative Rac1 prevented the augmentation of cofilin phosphorylation at the site of Ser3 induced by 50 µM NaHS, but it failed to blunt the activation of ERK triggered by NaHS (Fig. 8A, B). Similarly, RNA interference of Rac1 significantly decreased the elevated phosphorylation level of cofilin that was induced by 50 µM NaHS, whereas it had no effect on NaHS-induced elevation of phosphorylated ERK (Thr202/Tyr204) (Fig. 8C, D).

## Discussion

The process of endothelial cell migration is the main characteristic associated with blood vessel formation. Our previous study has shown that H_2_S is a proangiogenic factor both in vivo and in vitro [18], [19]. Cell migration is driven by the mechanical force provided by dynamic remodeling of the actin cytoskeleton which is regulated by several signaling pathways including members of the Rho GTPases family [23]. Here, we demonstrate that in human endothelial cells, H_2_S selectively activates the Rho GTPase Rac1, which in turn inactivates its effector molecule cofilin, inducing lamellipodia formation. The presented data also support that VEGFR-PI3K activates the Rac1-cofilin pathway and that H_2_S-induced Rac1 activation is specifically dependent on the p110α isoform of PI3K. These data provide the first evidence that the H_2_S-promoted migration of endothelial cells is dependent on Rac1-mediated actin cytoskeleton remodeling and enhance our understanding of the intracellular signaling mechanisms of the small molecule, H_2_S.

Importantly, the present study indicates that H_2_S induces the formation of typical lamellipodia in endothelial cells, suggesting a rearrangement of the actin cytoskeleton. While actin remodeling has an important role in driving cell migration [25]. Actin polymerization promotes protrusions named lamellipodia in the front edges of the migrating cells [26]. In the present research, the formed lamellipodia induced by H_2_S probably initiated the movement of the human endothelial cell.

Of all the signaling molecules that are involved in cell motility, Rho GTPases play a vital role in controlling actin dynamics [27]. Our evidence strongly demonstrates that Rac1 can be selectively activated by H_2_S, whereas we could not find evidence of RhoA or Cdc42 activated by H_2_S in the experiment. In the present study, the finding that dominant negative plasmid of Rac1 decreased the formation of lamellipodia suggests that Rac1 mediates the H_2_S-induced reorganization of the actin cytoskeleton. Therefore, we propose that the activation of Rac1 is essential for cell lamellipodia formation provoked by H_2_S.

A novel finding of the present study is the observation that Rac1 mediate the promoted cell migration effect induced by H_2_S in human endothelial cells, which is requisite for the angiogenesis. In the present study, suppression of Rac1 expression by RNA interference and inhibiting the endogenous activity of Rac1 by transfecting dominant negative plasmid both blunted the H_2_S-induced promoted migration effect of endothelial cells. Furthermore, the enhanced tube formation effect of endothelial cells by H_2_S was also greatly decreased. Moreover, the lamellipodia formation induced by H_2_S was revoked by Rac1 activity inhibiting. These data provide us with the first piece of evidence that the promoted migration effect of H_2_S on human endothelial cells is dependent on Rac1 mediated actin cytoskeleton remodeling.

H_2_S, a small, ubiquitous, gaseous, and diffusible molecule, can exert active properties in biological systems. Studies by Mustafa et al in the year of 2009 demonstrated that H_2_S can physiologically S-sulfhydrate proteins and that this sulfhydration modification of cysteine residues enhances the molecular activity of these proteins [28]. In our cell-free system, however, the observation that Rac1 was not directly activated by H_2_S gives us assured implication that Rac1 activation was potentially mediated by an upstream signaling molecule.

To date, the mechanisms that regulate the activity of Rho GTPases during cell migration have not been studied extensively. Rac1 activation by receptor tyrosine kinases usually depends on PI3K activity, and inhibitors of PI3K can block Rac1 activation [29], [30]. In the present study, pharmacologic inhibition of VEGFR and PI3K blunted the activated Rac1 and promoted cell migration induced by H_2_S, which gives us implication that VEGFR-PI3K pathway lay upstream of Rac1 and mediate the promoted cell migration effect of H_2_S. The observation that dominant negative Akt had no effect on the elevated activation of Rac1 induced by H_2_S indicates that H_2_S-induced Rac1 activation is independent of Akt.

PI3Ks has a pivotal role in the regulation of cell growth, cell survival, and cell motility [31]. Class I PI3Ks have been classified into class IA and class IB. Class IA contains three catalytic isoforms, i.e. p110α, p110β, and p110δ and class IB contains the p110γ isoform [32]. Previous studies have shown that angiogenesis selectively requires the p110α isoform of PI3K to control endothelial cell migration [33]. Researchers have also demonstrated that the activation of Rac1 is selectively dependent on the p110α isoform of PI3K [34]. In our study, the depletion of p110α using siRNA significantly inhibited the activation of Rac1 by H_2_S, whereas p110β siRNA, p110γ siRNA and p110δ siRNA had no effect on the activation of Rac1. Moreover, only depletion of p110α significantly inhibited H_2_S-induced cell migration. Taken together, these results suggest that H_2_S-induced Rac1 activation and cell migration are specifically dependent on the p110α isoform of PI3K.

With respect to mitogen-activated protein kinases (MAPKs), ERK can also regulate the activity of Rac1, allowing the formation of cellular protrusions and thereby contributing to tumor cell motility and invasion [35]. Studies have also demonstrated that ERK mediates the action of EGF by activating Rac1 to regulate cell migration [36]. But our results that inhibiting the activation of ERK has no effect on the activated Rac1 induced by H_2_S strongly suggest that Rac1 activation by H_2_S is independent of ERK.

Rac1 activates PAK which subsequently stimulates LIM-kinase (LIMK). Activated LIMK can inactivate cofilin by inducing phosphorylation of cofilin, while non-phosphorylated cofilin can induce actin depolymerization [37]. In this context, Rac1 has the ability to inhibit cofilin-induced actin depolymerization. There is also strong evidence that cofilin is required for and promotes lamellipodia extension and cell migration [38]. In our study, the phosphorylation level of cofilin, which was stated as inactive form, elevated in a time-dependent manner after H_2_S treatment. Besides, suppression of Rac1 expression by RNA interference and inhibiting the endogenous activity of Rac1 by transfecting dominant negative plasmid both abolished the augmented phosphorylation level of cofilin. Moreover, pharmacologic inhibition of the VEGFR-PI3K pathway significantly attenuated the level of phosphorylated cofilin. Based on these observations, we validate that cofilin acts as a downstream effector of Rac1 and may take its effect to modulate actin dynamics triggered by H_2_S. Whether there exist other downstream effectors still needs further investigation.

**Figure 9:**
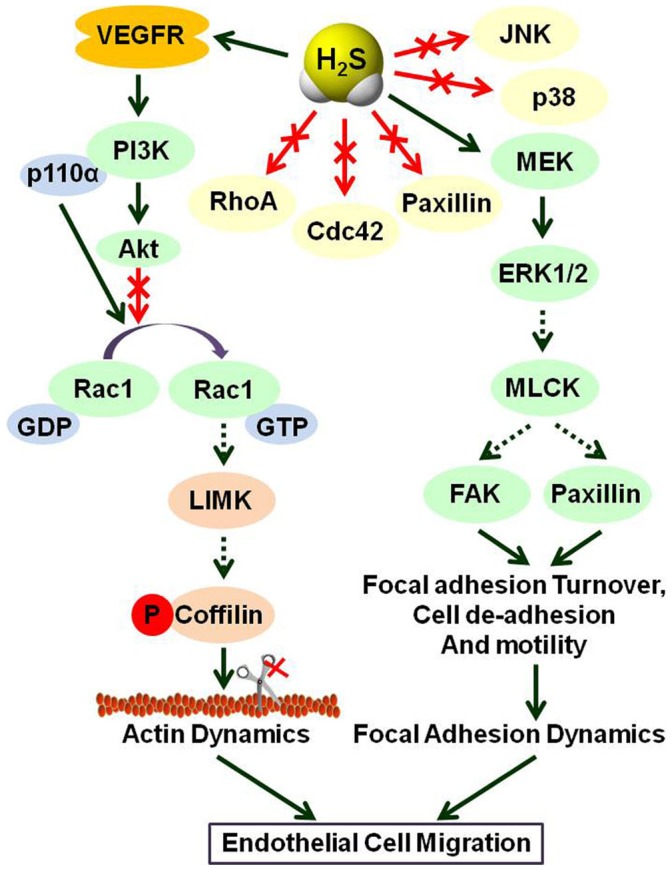
Model illustrating the underlying signal transduction pathways of H_2_S that affect the migration of endothelial cells.

It is well established that MAPKs, involving p38, ERK and Jun N-terminus kinase (JNK), have pivotal roles in the regulation of cell migration via various signaling pathways. P38 regulate cell migration by inducing phosphorylation of MAPK-activated protein kinase 2/3 (MK 2/3) and subsequent phosphorylation of HSP27 which is involved in directional migration of the cell [39], [40]. ERK has been shown to regulate cell motility by inducing phosphorylation of myosin light chain kinase (MLCK) [41], paxillin [42] or FAK [43]. While JNK regulates cell migration by inducing phosphorylation of paxillin [44]. In our present study, we observed transient phosphorylation level elevations on ERK. Neither JNK nor p38 were under phosphorylated changements after NaHS treatment. And the finding that inhibition of MEK-ERK markedly reduced the migrated cell number that was augmented by H_2_S indicates that MEK-ERK pathway partially mediates the promotion of cell migration caused by H_2_S. ERK has also been found to be a downstream regulator of Rac1 in the regulation of cell motility [45]. But our evidence that the ERK phosphorylation caused by H_2_S was not inhibited by Rac1 suppression, indicating that the action of ERK on cell migration is independent of the Rac1 pathway. Previous data have shown that ERK activation play an important role in the regulation of actin-myosin mediated contraction [46]. MLCK (MLC kinase) can be directly activated by ERK and thereby leading to the phosphorylation of myosin light chains, which is important for the key processes in cell migration including the formation of adhesion complexes and focal adhesions [47]. Accordingly, we speculate that ERK may affect endothelial cell motility by its substrates (eg. MLCK ) to modulate focal adhesion dynamics. On the other hand, our data demonstrate the pathway of VEGFR/PI3K/Rac1/cofilin mediate the H_2_S-induced endothelial cell migration through actin cytoskeleton remodeling. Taken together, we conclude that the endothelial cell migration induced by H_2_S seems to be independently mediated through the pathway of VEGFR/PI3K/Rac1/cofilin and MEK-ERK, which suggest their different roles in the action of H_2_S. However, the exact role of the two respective signaling pathway in H_2_S-promoted endothelial cell migration should be further investigated.

In conclusion, H_2_S signals through the VEGFR-PI3K pathway to activate Rac1 and thus inactivates the downstream effector cofilin, which in turn influences actin dynamics by triggering the formation of lamellipodia. In addition, our data support that H_2_S-induced Rac1 activation is specifically dependent on the p110α isoform of PI3K. These observations demonstrate that H_2_S activates Rac1 in a PI3K p110α-dependent fashion and ascribes a novel function to H_2_S in the regulation of the actin cytoskeleton and cell migration. The induction of the VEGFR/PI3K/Rac1/cofilin pathway by H_2_S contributes to the regulation of endothelial cell motility via actin cytoskeleton reorganization. Moreover, the MEK-ERK pathway is also involved in the effect of H_2_S on endothelial cell migration but is independent of Rac1. MEK-ERK pathway may play its effect through focal adhesion dynamics (Fig. 9).
